# Mortality in Transition: Study Protocol of the PrivMort Project, a multilevel convenience cohort study

**DOI:** 10.1186/s12889-016-3249-9

**Published:** 2016-07-30

**Authors:** Darja Irdam, Lawrence King, Alexi Gugushvili, Aytalina Azarova, Mihaly Fazekas, Gabor Scheiring, Denes Stefler, Katarzyna Doniec, Pia Horvat, Irina Kolesnikova, Vladimir Popov, Ivan Szelenyi, Michael Marmot, Michael Murphy, Martin McKee, Martin Bobak

**Affiliations:** 1 University of Cambridge, Cambridge, UK; 2 London School of Economics, London, UK; 3 Department of Epidemiology and Public Health, University College London, 1-19 Torrington Place, London, WC1E 6BT UK; 4 Institute of Economics of the National Academy of Sciences of Belarus, Minsk, Belarus; 5 New Economic School, Moscow, Russia; 6 Yale University, New Haven, USA; 7 London School of Hygiene and Tropical Medicine, London, UK

**Keywords:** Mortality, Privatization, Post-communist transitions, Multi-level analysis

## Abstract

**Background:**

Previous research using routine data identified rapid mass privatisation as an important driver of mortality crisis following the collapse of Communism in Central and Eastern Europe. However, existing studies on the mortality crisis relying on individual level or routine data cannot assess both distal (societal) and proximal (individual) causes of mortality simultaneously. The aim of the PrivMort Project is to overcome these limitations and to investigate the role of societal factors (particularly rapid mass privatisation) and individual-level factors (e.g. alcohol consumption) in the mortality changes in post-communist countries.

**Methods:**

The PrivMort conducts large-sample surveys in Russia, Belarus and Hungary. The approach is unique in comparing towns that have undergone rapid privatisation of their key industrial enterprises with those that experienced more gradual forms of privatisation, employing a multi-level retrospective cohort design that combines data on the industrial characteristics of the towns, socio-economic descriptions of the communities, settlement-level data, individual socio-economic characteristics, and individuals’ health behaviour. It then incorporates data on mortality of different types of relatives of survey respondents, employing a retrospective demographic approach, which enables linkage of historical patterns of mortality to exposures, based on experiences of family members. By May 2016, 63,073 respondents provided information on themselves and 205,607 relatives, of whom 102,971 had died. The settlement-level dataset contains information on 539 settlements and 12,082 enterprises in these settlements in Russia, 96 settlements and 271 enterprises in Belarus, and 52 settlement and 148 enterprises in Hungary.

**Discussion:**

In addition to reinforcing existing evidence linking smoking, hazardous drinking and unemployment to mortality, the PrivMort dataset will investigate the variation in transition experiences for individual respondents and their families across settlements characterized by differing contextual factors, including industrial characteristics, simultaneously providing information about how excess mortality is distributed across settlements with various privatization strategies.

**Electronic supplementary material:**

The online version of this article (doi:10.1186/s12889-016-3249-9) contains supplementary material, which is available to authorized users.

## Background

The first half of the 1990s witnessed an estimated 7 million excess premature deaths in post-communist countries, coinciding with large socio-economic transitions. Subsequent major economic fluctuations, such as the 1998 [1] and the 2007 crises, caused significant spikes in mortality [2]. Epidemiological research has uncovered various proximal causes of these deaths, identifying alcohol and social and psychological stress as the key reasons for increased mortality [1–4]. However, the more distant factors remain less clear. Earlier work in Russia pointed to the role of rapid transition, as reflected by employment turnover [5, 6]. More recently, an extensive cross-national time-series analysis found an association between different privatization approaches and increased mortality [7]. However, previous studies on the post-communist mortality crisis have relied on individual-level studies or routine data; such studies cannot assess both distal (societal) and proximal (individual) causes simultaneously, and are therefore unsuitable for investigating the pathways linking societal factors with mortality.

The PrivMort project was designed to address these research drawbacks by linking micro- and macro-level data and determining the hierarchy of causes of mortality, from the distal (such as privatization strategy) to the proximal (e.g. alcohol consumption) in three post-communist countries (Russia, Belarus and Hungary).

## Methods and design

### Aims of the project

The PrivMort is a multi-disciplinary project in the subfield of the Political Economy of Public Health, funded by the European Research Council. The project’s main research objectives are: 1) to conduct an in-depth investigation into the post-Communist mortality crisis using multi-level data; 2) provide meso- and micro-data to test the privatization-mortality hypothesis; 3) to understand whether post-Communist mortality in general and any potential privatization-induced mortality in particular are influenced by social factors such as class, occupational position, education, gender and community-level factors; and finally, 4) to examine the effect of these social variables on health outcomes in the post-communist states.

### Design and setting of the study

The PrivMort project is based on analysis of two large-scale datasets. The first is a set of sample surveys conducted in 30 settlements in Russia, 20 in Belarus and 52 in Hungary. The second is a set of annual time series covering the period 1990–2010 for 539 settlements in Russia, 96 settlements in Belarus, and 52 settlements in Hungary, which include the settlements that the sample surveys were conducted in. These datasets are described below.

### Indirect demographic convenience cohort

The multi-level retrospective convenience cohort study uses a demographic approach, originally designed for estimating mortality in countries without vital registration systems [8]. This method collects information from informants in censuses or sample surveys about the survival status of a range of their different relatives that can provide information on mortality across a wide range of different cohorts. In the PrivMort project, interviews with randomly selected respondents collected information on their relatives’ mortality in years spanning the period of transition. The study has been approved by the University of Cambridge Department of Sociology ethics committee. All data are anonymized to prevent any potential identification of individual respondents.

The method was originally used in countries with low levels of literacy and numeracy where only very simple questions such as “Is your mother alive?” could be used. Such questions have been validated and are recommended by the United Nations for use in censuses. With appropriate modelling, this information may be used to produce standard indicators such as age-specific mortality rates. Since this information was not collected directly, but inferred, these methods that were developed particularly by William Brass and colleagues are often referred to as “indirect estimation” or “Brass techniques”. In cases where vital registration systems exist, the underlying method of asking informants about their relatives’ mortality can provide information on topics that could not realistically be obtained in any other way. One example is educational differences in mortality in Russia. Since 1997, educational level is no longer collected on death certificates, so routine information is no longer available, but estimates can be made by collecting information form respondents on educational level and survival of their relatives. In highly literate and numerate societies, information on age and date of death can be collected so that more refined estimates may be made. Since we are particularly interested in events occurring over 20 years ago in specific locations and including information on topics such as individuals’ employment histories and smoking and drinking, no alternative source of information actually exists.

### Settings

The PrivMort project used a multilevel approach by sampling mono-industrial towns with radical privatization and then matching them with towns with slow privatization experiences. This approach provides an opportunity to estimate the effect of privatization on health, while controlling for individual-level confounding factors. Mono-industrial towns were defined as where a single industrial enterprise provided employment for at least 7.5 % of the total population in 1991, while the second large industrial enterprise employed less than 2.5 % of the total population (see for example [9–11]). The Russian towns were selected from those with 5,000–100,000 inhabitants in the European part of the country, excluding those that were within 50 km to Moscow and St. Petersburg (*N* = 539) (see Additional file 1). The Belorussian towns were selected from those with 5,000–100,000 inhabitants, excluding those within 50 km from Minsk (*N* = 96). The Hungarian towns were selected from those with 5,000–100,000 inhabitants that were not located in Pest county (*N* = 110).

In Russia 10 mono-industrial towns with fast privatization were matched with 10 mono-industrial towns with slow privatization, (fast privatized towns are towns, where 90 or more per cent of state shares were privatized within two consecutive years, and slow privatized towns are towns in which less than 50 % of state shares were privatized within two consecutive years.) After that, a matching control group of four multi-industrial towns with fast privatisation and one multi-industrial town with slow privatization were selected. In Hungary 52 towns were selected at random from amongst the industrial towns (*N* = 83) in the original list of 110 towns. We chose this strategy mainly to address the far greater diversity of privatisation strategies applied in Hungary.

There are 11 mono-towns in Belarus in total, so all of them were included into the dataset. After that, 9 multi-slow towns in Belarus were matched to the 15 mono-fast towns selected in Russia. The full list of settlements can be found in the Additional file 2. Towns were matched using standard propensity score matching based on the pre-transition demographic and socio-economic conditions in the settlements. We used eight potential predictors of mortality levels, all measured for the pre-transition year, 1991, with the exception of wage in USD, which was available from 1992: (1) crude death rates per 1000 population in 1991; (2) pre-reform population size, (3) dependency ratio in 1991; (4) average wage in US dollars in 1992; (5) number of physicians per 10,000 population in 1991; (6) floor area per person in 1991; (7) death rates from alcohol poisoning per 100,000 population in 1991; and (8) emission of pollutants into the atmosphere from stationary sources, thousand tons in 1991.

In Russia, for example, this means that we had two groups of mono-industrial towns that were close to identical on these 8 variables, but one group experienced fast privatization and one group slow privatization. The same procedure was employed to match multi-industrial towns to mono-industrial towns.

In Russia, the settlement-level database was based on the Economy of Russian Cities database provided by the Main Interregional Centre for Processing and Dissemination of Statistical Information of the Federal State Statistics Service (GMC Rosstat). The data on the population of the settlements came from the historical state dataset on Russian towns since 1897. The enterprise-level data were collected from a variety of state sources. In Russia many enterprises changed their registry code after privatization, which required a rigorous selection process. First, enterprises with less than 500 employees were eliminated from the dataset in Russia. Then, the datasets were matched based on the OKPO (National Classification of Enterprises and Organizations by the State Statistics Service) registration code for enterprises, while for those firms that changed their code after being privatized, the matching was based on the address, enterprise name and the approximate size of the enterprise within a settlement in some cases.

In Belarus the settlement-level data were acquired through the National Statistical Committee of the Republic of Belarus (Belstat); from various regional bodies of the National Statistical Committee; from the Ministry of Health of the Republic of Belarus; and from the Ministry of Internal Affairs. The enterprise-level data for Belarus were collected from the Bureau van Dijk; from National Statistical Committee of the Republic of Belarus (Belstat); and from the Unified State Registry of Legal Entities including the State Property Committee of the Republic of Belarus; and JSC Belarusian Currency and Stock exchange.

The Hungarian settlement-level data were obtained from the Hungarian Central Statistical Office and a variety of other government sources. The largest companies in the selected settlements based on registered capital in 1990 were identified through the Company Information Service of the Ministry of Justice. Some data were acquired from the Hungarian Privatization Agency. After selecting the three biggest companies in each settlement data on the number of employees, on ownership structure and profitability were collected from the archives of the local courts of registry and from various private digital company information archives. Most of the companies that existed in 1990 changed their names or their legal form. To ensure continuity the successors of the original parent companies were identified and data on them were obtained. Overall, altogether we collected data on 383 Hungarian companies. For analytical reasons, original parent companies and successors were later treated as one company. When there was more than one successor company, the biggest company by registered capital or the one closest to the original company by type of activity was selected.

### Participants in population surveys

A random walk procedure was used in the PrivMort for sampling the respondents. First, the settlements were divided into street-centered clusters, which were then distributed among the interviewers using the method of random numbers. Each route could generate up to 25 interviews. Starting from the first house on the street in each cluster, a step of four was applied in private houses and in apartment blocks.

Only one respondent was selected from each household, even in cases when more than one family shared the same house. In cases where more than one person in the household matched the screening criteria (older than 42 years of age; relatives lived in the same settlement between 1980 and 2010), the person whose birthday is closer to the date of the survey was selected for the interview. Interviewers had to make four attempts at interviewing the person who matched the screening criteria if he or she was temporarily unavailable.

All respondents were born before 1972 to ensure that they and their relatives were of working age in 1991 and hence could potentially be affected by the transition. The selection was conditional on the fact that their family members lived in the same settlement for a prolonged period of time during and after the transitions. While the criterion for women was to have at least one family member (parents, siblings or a spouse) living in the same settlement in this time period, the survey excluded male respondents who only had their spouses residing in the same settlement.

### Subjects of the convenience cohort

The convenience cohort consists of three types of relatives of the respondents in the population surveys: parents, siblings and spouses/partners. The survey uses the orphanhood method to obtain information about the respondents’ parents’ year of birth and whether they are alive or dead and, if so, when they died. Information on survival is collected for a maximum of two siblings who survived to age 20, the age at which our analysis of adult mortality starts. (The proportion of informants with larger number of siblings is small; for reasons of interview efficiency, additional information collected would be counter-productive, and more siblings would lead to even greater over-representation of those from large sibships). The third group of relatives consists of the first partners (married or long-term cohabiters) of female respondents. We ask about survival of the first partner, since if current partner were included, information on men who died early would be differentially excluded and therefore bias results. Since our main interest was in male mortality, we did not ask for information on female partners from male respondents.

### Collection of data

Data were collected in face-to-face interviews using structured questionnaires, covering characteristics of the respondents and their relatives, including the residency history (including some questions on international and domestic migration); education levels; marital status; religious affiliation; lifestyle habits such as smoking and alcohol consumption; vital status of relatives, a substantial separate block of questions on issues such as the labour market position and employment history; and some questions on economic conditions. The section containing questions on alcohol consumption is particularly detailed and contains measures and estimations of the frequency and the amount of drinking, of the character of drinking or the type alcohol consumed, and on the consumption of health-hazardous spirits. The list of areas covered by the questionnaire is shown in Table 1. The full questionnaire is available upon request. The questions and response categories varied slightly among three countries; the questions on alcohol are slightly different for Hungary as the types and amounts of alcohol consumed that are perceived as ‘normal’ vary between countries.

**Table 1:** List of domains examined by the questionnaire about members of the indirect cohort

Domain	Measures
Demographic information	Date of birth; gender; marital status; religion
Residential history	Residence places for the last 3 decades; reasons for moving
Socioeconomic status	Position; occupation; number of subordinates
Labour market history	Employment history for the last 3 decades; ISCO
Education	Highest level of education obtained
Health behaviours	Smoking; drinking (frequency; binge drinking; *zapoi*; hazardous drinking)
Material deprivation	Absolute poverty proxies
Ownership	Ownership of material resources
Social capital	Communication with relatives

The questionnaire was developed by a multidisciplinary team of researchers, followed by cognitive testing of questions on respondents sampled from mono-industrial towns using the snowball sampling. The cognitive tests were carried out in a controlled environment to identify problematic wording and sensitive questions. As a result of the cognitive interviews, the questionnaire has been modified to ensure a smooth flow of the conversation and to make the respondents as comfortable and confident as possible. There were then small pilot surveys conducted in each country before large scale surveying began. The interviews (taking approximately 50 min on average) were conducted in the respondents’ homes in the period from mid-September 2014 to March 2015 [9].

The data collection agencies responsible for conducting the interviews were required to perform back-checks for at least 10 % of the interviews conducted in each settlement and 15% of unsuccessful interview attempts. The back-checks were mostly performed randomly by phone, while in some cases the regional supervisors carried out the back-checks by visiting individual households.

During the cognitive tests, we discovered that respondent sensitivity was less of a problem than initially feared, consistent with previous experience in Russia where people often appreciated the opportunity to talk about their deceased relatives, knowing that they were contributing to research that may benefit public health in the future [12].

Total numbers of subjects and their distribution across countries and town types are shown in Table 2. Numbers of subjects in each settlement type are presented in Table 3.

**Table 2:** Numbers of respondents, their relatives and deaths among relatives, and response rates in the population surveys

Country (response rate)	Total	Dead	Dead (%)
Russia (48 %)			
Respondents	22 997	-	-
Relatives	71 009	36 560	51.5
- Fathers	16 268	11 585	71.2
- Mothers	19 870	13 231	66.6
- Siblings	18 910	5 467	28.9
- Partner/Husband	15 961	6 277	39.3
Total Subjects	94 006	-	-
Belarus (39 %)			
Respondents	16 000	-	-
Relatives	55 976	26 602	47.5
- Fathers	11 308	8 617	76.2
- Mothers	13 677	8 930	65.3
- Siblings	19 634	4 856	24.7
- Partner/Husband	11 357	4 199	37.0
Total Subjects	71 976	-	-
Hungary (85 %)			
Respondents	24 076	-	-
Relatives	78 622	39 809	50.6
- Fathers	15 803	12 728	80.5
- Mothers	19 521	13 194	67.6
- Siblings	28 764	8 115	28.2
- Partner/Husband	14 534	5 772	39.7
Total Subjects	102 698	-	-
All countries (58 %)			
Respondents	63 073	-	-
Relatives	205 607	102 971	50.1
- Fathers	43 379	33 260	76.7
- Mothers	53 068	35 709	67.3
- Siblings	67 308	18 438	27.4
- Partner/Husband	41 852	16 248	38.8
Total Subjects	268 680	-	-

**Table 3:** Numbers of subjects (deaths) by settlement type and country in the convenience cohort restricted to relatives who were in working age in 1992

	Russia	Belarus	Hungary
Town type	N (deaths)	N (deaths)	N (deaths)
Mono-Industrial Fast	35 322 (18 603)	-	-
Mono-Industrial Slow	24 333 (12 607)	23 048(8 380)	-
Multi-Industrial, Fast and Slow	7 414 (3 349)	19 008 (6 826)	-
Total	67 069 (34 559)	42 056(15 206)	56 143(20 138)

### Additional surveys

PrivMort is currently (spring and summer 2016) conducting a new set of surveys. First, 1,500 representative interviews will be conducted in both the European part of Russia and in Hungary. This will significantly increase the generalizability of the findings. In addition, 8,800 interviews containing a set of questions on self-reported health and psychological wellbeing will be collected in Russia. All of these interviews will be collected in 23 randomly selected towns in Sverdlovsk Oblast - one of the most typical industrial regions of the European part of Russia, where the mortality trends on average match the country averages perfectly. Finally, the PrivMort project conducts qualitative fieldwork in Russia and Hungary in order to compliment the surveys. The qualitative interviews will be developed around a set of semi-structured interviews, mostly covering people’s perceptions of the transitions. The interviews intend to also address the issues stemming from the unaccountable domestic migration, which might have not been captured by the sampling design.

### Statistical analysis

A number of studies have combined macro-level time series for different geographical units with individual-level survey data from sources such as SHARE, HRS and EU-SILC [13] to identify the effect of variables such as unemployment rates or retirement age and micro-data on individuals’ wellbeing and mortality [14, 15]. For reasons noted above, relevant individual-level data do not exist, but we have shown that we can use reports for relatives as an alternative data source [3, 16–18].

Our data permit the allocation of each mortality event to a cell with a specific set of characteristics, such as, for instance, the number of deaths among well-educated men age 45 in a rapid-privatization town in 1994. The corresponding person-years of exposure to risk in the group may be calculated using standard software (e.g. the pyears function in R 3.2.2). Mortality rates may be fitted using a standard Poisson GLM with logarithm of exposure as offset, with both individual and macro-level data as covariates.

The model coefficients may be used to show the relative risks associated with various covariates, and the fitted values of mortality rates may be used to construct variables such as life expectancy at age 40 for an individual with a specified set of characteristics or variables such as the probability of survival from 20 to 65.

These data may be used to fit a number of alternative survival models including Cox regression models, with or without time-dependent covariates, or parametric models such a Gompertz or Weibull distributions. The data have a multi-level structure, with subjects (i.e. relatives) nested within families, within towns and finally within countries. These structures can be modeled using multi-level models. Since there is a rich settlement-level macro-level dataset, these variables can be included in such models where appropriate.

## Discussion

The PrivMort project investigates the distal and proximal causes of mortality in post-communist transition countries. To achieve this ambitious objective, the project established a retrospective indirect convenience cohort of relatives of respondents in population surveys. These individual level-data are complemented by settlement level data from other sources.

The complex methodology has several limitations. First, it was not possible to cover the whole post-communist region. We focus on the European part of Russia and Belarus mainly because the post-Communist mortality crisis was especially severe in these regions of the Former Soviet Union. These two former Soviet countries are relatively homogeneous, with similar religious, cultural and socio-economic characteristics but different pace and type of privatization, which makes the comparison especially favourable, as it allows us to test for overall transition strategy at the country level. The population survey only covers the European part of Russia; however, about 70 % of Russia’s total population lives in the European part of the country, which makes data collection in this region reasonable in terms of costs and generalisation. Excluding the region of the North Caucasus from the sample is crucial as the region was torn by several conflicts in the 1990s and 2000s. Including Hungary, which applied a gradual privatization model with a significantly higher share of foreign ownership [19], makes it possible to investigate the same issues in a non-Soviet country.

Second, the convenience cohort based on the Brass method is not directly representative of a defined population and the chance of inclusion is not uniform. However, since we obtain information from a range of different types of individuals, very few people are excluded (an older only child unmarried woman whose parents are dead would be an example). However, there is no evidence that this would lead to biases that would invalidate our findings given that we are concerned with adult mortality in a developed country, even though concerns have been raised about such issues for infant and toddler mortality in least-developed countries [20]. A similar issue relates to the modest response rates. As non-respondents tend to have lower socioeconomic status and worse health, and since their relatives are likely to be of similar background and have similar health status, the convenience cohort is likely to be less well-off and less healthy than the general population. This, however, should not affect the associations within the study. In addition, the fact the cohort is formed from respondents’ relatives, rather than respondents themselves, further reduces the potential effect of the low response rates on the results.

Third, an additional potential source bias relates to family-level factors. Subjects (relatives) who left the selected settlement area were excluded from the study, so future investigations should look into the migrant differentials on the settlement level. However, if there was a strong family-level mortality effect on migration, this would lead to high mortality families being less likely to have any surviving relative alive in 2015 to report on their mortality two decades earlier, which would differentially exclude high mortality families. Analysis of family-level clustering on earlier data sets suggests that this is not a major source of bias, but further work will be undertaken. We have noted some of the possible concerns about the use of such an approach, but we emphasize that under reasonable assumptions the method produces largely unbiased estimates of mortality that can be directly compared with other sources such as official statistics.

Fourth, recall bias and measurement error can be a major issue in a cohort where all the data come from proxy informants (i.e. relatives). Such misclassification leads to underestimation of the strengths of associations between risk factors and mortality and reduce the statistical power of the study. However, this is at least partly compensated by the large size of the study (although we are aware that the scale of such bias depends on the magnitude of misclassification of the variables of interest). In addition, to further reduce misclassification, only male spouses were included in the convenience cohort, as the literature suggests that men are more likely to exclude non-residential: former, and especially first, partners in surveys. Women are more likely to report better on non-residential partners due to social pressures, cultural perceptions and other factors (see for example [3]).

Fifth, parents’ mortality tends to be underreported by the respondents if information is provided on adoptive rather than dead natural parents [21, 22]. To address this potential problem, the survey specifically asks respondents raised by adoptive parents, to respond to the questions about their biological parents, wherever possible. Another danger of the orphanhood method is that in general the chance of an individual being included is proportional to the number of living children, and completely excludes people who have failed to produce any offspring or had no surviving children [23]. However, evidence form developed countries suggests that mortality differentials according to number of children are small and therefore unlikely to bias results. As with parents, the reported siblings are not uniformly distributed. Those from large sibships will have more chances of being included and only children have zero chance of being included. Mortality among infants and children has been found to be much higher in some situations such as in developing countries, due to reasons such as the harmful effects of short birth intervals. However, this is of minor importance for this study, because we are concerned with adult mortality and there is no evidence of strong sibship size effects on mortality; in any case, large sibships in these populations are uncommon.

Sixth, the literature indicates that events occurring years ago tend to be misreported or reported with a limited degree of accuracy in surveys [24]. Since PrivMort is based on the retrospective data, it was fundamental for the project to address this limitation. Some techniques such as introductory sentences before question sections have been recommended in the literature as significantly increasing the recall precision in respondents [25]. Also, the way that questions are framed has been demonstrated to affect the recall bias in surveys [26]. This was considered in the development of the questionnaire, where for some retrospective questions especially on the behaviour of the relatives we added proxy questions. For instance, since it might sometimes be challenging for respondents to recall whether or not their fathers would drink extensively not least because they might not be communicating directly, the questionnaire contains a proxy question “Have any of your family members of friends seen your father drunk?”, based on our earlier experience in conducting research on alcohol in Russia [12]. Also, since using some memorable events that remind the respondents of their life and behaviour years ago been proven as a successful way of addressing the recall bias in retrospective surveys [27], the PrivMort questionnaire includes some historical events, such as the collapse of the Soviet Union, for example, framed inside the questions. Furthermore, for particularly complex questions, or questions requiring the respondents to recall some life events occurring decades ago, the interviewers were handed response cards, which depicted the response options or outlined the time periods in question.

We are aware of these limitations of the indirect cohort approach, but we are confident that under reasonable assumptions the method produces unbiased estimates of mortality that can be directly compared with other sources such as official statistics.

## Conclusion

Focusing on different combinations of micro-, meso- and macro-level variables, the PrivMort project seeks to uncover individual health behaviours, adaptations and preferences and their effect on mortality during societal transformation. The methodology of the PrivMort project provides a useful and flexible model for interdisciplinary research in post-Communist countries and elsewhere.

## Additional file

Additional file 1: The Location of the PrivMort Towns on The Map of The European Part of Russia. (DOC 1105 kb) Additional file 2: The Full List of Settlements Used in The PrivMort Project. (DOC 32 kb)
