# Supplementary material for: Mortality in Transition: Study Protocol of the PrivMort Project, a multilevel convenience cohort study
Source: BMC Public Health. 2016 Jul 30;16:672. doi: 10.1186/s12889-016-3249-9 (PMC4967292; doi:10.1186/s12889-016-3249-9)
Supplement: Additional file 1: — The Location of the PrivMort Towns on The Map of The European Part of Russia. (DOC 1105 kb) [file 12889_2016_3249_MOESM1_ESM.doc]

**Additional File 1**

**
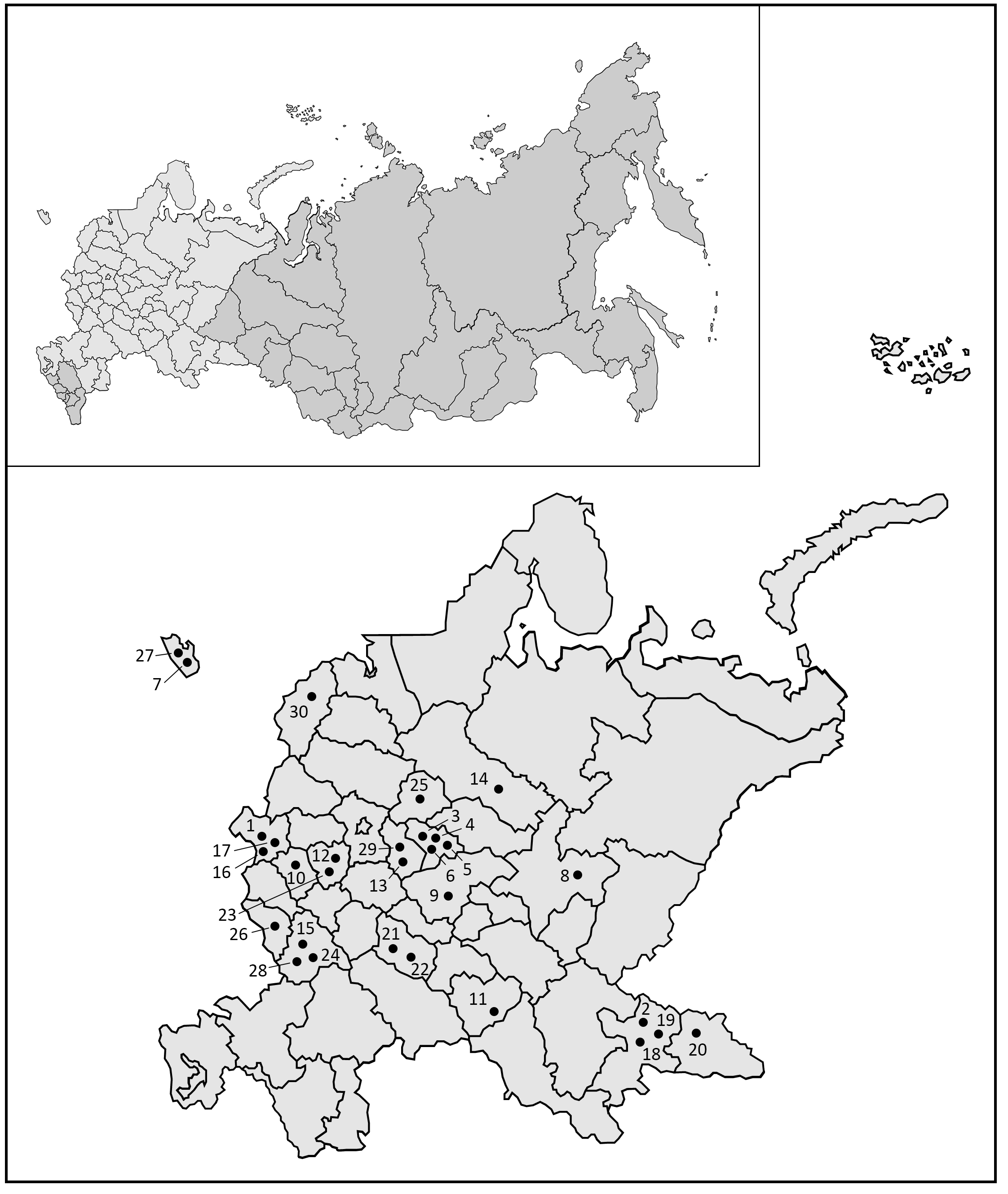
**

Note: 1. Zhukovka; 2. Karabash; 3. Kohma; 4. Navoloki; 5. Privolzhsk; 6. Yuzha; 7. Nieman; 8. Kirov-Chepetsk; 9. Kulebaki; 10. Mtsensk; 11. Otradny; 12. Yasnogorsk; 13. Lakinsk; 14. Nikolsk; 15. Semiluki; 16. Seltso; 17. Starodub; 18. Bahcall; 19. Sim; 20. Dalmatovo; 21. Belinsky; 22. Nikolsk; 23. Plavsk; 24. Boguchar; 25. Danilov; 26. Alekseevka; 27. Svetlyj; 28. Buturlinovka; 29. Moorom; 30. Pechora.
