# Supplementary material for: Mortality in Transition: Study Protocol of the PrivMort Project, a multilevel convenience cohort study
Source: BMC Public Health. 2016 Jul 30;16:672. doi: 10.1186/s12889-016-3249-9 (PMC4967292; doi:10.1186/s12889-016-3249-9)
Supplement: Additional file 2: — The Full List of Settlements Used in The PrivMort Project. (DOC 32 kb) [file 12889_2016_3249_MOESM2_ESM.doc]

**Additional File 2**

**a. Settlements in The Russian Sample**

Zhukovka Bryanskaya oblast’

Karabash Chelyabinskaya oblast’

Kohma Ivanovskaya oblast’

Navoloki Ivanovskaya oblast’

Privolzhsk Ivanovskaya oblast’

Yuzha Ivanovskaya oblast’

Nieman Kaliningradskaya oblast’

Kirov-Chepetsk Kirovskaya oblast’

Kulebaki Nizhegorodskaya oblast’

Mtsensk Orlovskaya oblast’

Otradny Samarskaya oblast’

Yasnogorsk Tulskaya oblast’

Lakinsk Vladimirskaya oblast’

Nikolsk Vologodskaya oblast’

Semiluki Voronezhskaya oblast’

Seltso Bryanskaya oblast’

Starodub Bryanskaya oblast’

Bahcall Chelyabinskaya oblast’

Sim Chelyabinskaya oblast’

Dalmatovo Kurganskaya oblast’

Belinsky Penzenskaya oblast’

Nikolsk Penzenskaya oblast’

Plavsk Tulskaya oblast’

Boguchar Voronezhskaya oblast’

Danilov Yaroslavskaya oblast’

Alekseevka Belgorodskaya oblast’

Svetlyj Kaliningradskaya oblast’

Buturlinovka Voronezhskaya oblast’

Tikhoretsk Krasnodarskij kraj

Pechora Pskovskaya oblast’

**b. Settlements in The Hungarian Sample**

Baja

Kiskunhalas

Kalocsa

Komló

Békéscsaba

Szeghalom

Edelény

Szerencs

Alsózsolca

Csongrád

Dunaújváros

Sárbogárd

Sopron

Mosonmagyaróvár

Kapuvár

Hajdúböszörmény

Hajdúhadháza

Kaba

Eger

Szolnok

Jászberény

Törökszentmiklós

Karcag

Tiszafüred

Kisújszállás

Kunszentmárton

Kunhegyes

Martfű

Oroszlány

Dorog

Kisbér

Lábatlan

Salgótarján

Kaposvár

Nagyatád

Tab

Kisvárda

Nyírbátor

Vásárosnamény

Szekszárd

Paks

Szombathely

Sárvár

Celldömölk

Kőszeg

Ajka

Berhida

Zalaegerszeg

Nagykanizsa

Lenti

Satoraljaujhely

Batonyterenye

**c. Settlements in The Belarus Sample**

Berezovka

Buda-Koshelevo

Kamenets

Khojniki

Kostyukovichi

Krasnosel’skij

Lepel’

Lyakhovichi

Mikashevichi

Mosty

Nesvizh

Orsha

Oshmyany

Polotsk

Schuchin

Slonim

Soligorsk

Svetlogorsk

Volkovysk

Zel’va
